# The association between smoking cessation and lifestyle/genetic variant rs6265 among the adult population in Taiwan

**DOI:** 10.1038/s41598-023-48806-x

**Published:** 2023-12-18

**Authors:** Yi-Ling Lai, Connie Cai Ru Gan, Oswald Ndi Nfor, Wen-Yu Lu, Shiuan-Shinn Lee, Yung-Po Liaw

**Affiliations:** 1 Department of Public Health and Institute of Public Health, Chung Shan Medical University, Taichung, 40201 Taiwan; 2 Community Health Center, Taichung Tzu Chi Hospital, Buddhist Tzu Chi Medical Foundation, Taichung, 427213 Taiwan; 3 Centre for Environment and Population Health, School of Medicine and Dentistry, Griffith University, Nathan, QLD Australia; 4 Institute of Medicine, Chung Shan Medical University, Taichung , 40201 Taiwan; 5 Department of Medical Imaging, Chung Shan Medical University Hospital, Taichung, 40201 Taiwan

**Keywords:** Genetics, Risk factors

## Abstract

Recent studies showed significant associations between socio-demographic, lifestyle factors, polymorphic variant rs6265, and smoking cessation behaviours. We examined rs6265 TT, TC and CC genotypes and their association with socio-demographic and other variables, including mental health status, drinking, exercise, and smoking behaviour among Taiwanese adults. Data on rs6265 were retrieved from the Taiwan Biobank, which contained genetic data collected between 2008 to 2019 from 20,584 participants (aged 30–70 years). Participants who smoked for more than 6 months prior to enrolment were categorized as smokers. If they had smoked and later quit for more than 6 months, they were classified as former smokers. Information regarding drinking, exercise, depression, and bipolar disorder were obtained through questionnaires and were categorized as either as affirmative (yes) or negative (no) responses. In contrast to previous studies, we found that the association between the polymorphism rs6265 and smoking behaviour was not significant (*P*-value = 0.8753). Males with lower education levels, young persons, and alcohol drinkers showed significant smoking behaviours (*P*-value < .0001). This population-based study indicates that rs6265 has no significant correlations with smoking cessation behaviour among adults in Taiwan.

## Introduction

The World Health Organization (WHO) estimated that smoking caused 2.1 million deaths and 13.6 million years lost to disability (YLDs) among adults in 2021^1^. Smoking-related diseases are a major cause of death among working-age adults, accounting for nearly 12% of all deaths worldwide^2^. Although the proportion is highest in Europe and the Americas^2^, Asia is the world’s largest tobacco consumer^3^. Guidelines suggest that smoking cessation programs should be tailored to sub-populations with behavioural support and pharmacotherapy. However, a recent study showed that the association between the genetic profile of an individual and smoking behaviour could help improve understanding and enhance smoking cessation intervention for those with a specific genetic variant. To date, there have been limited studies on Asians regarding the association between smoking cessation behaviour and the genetic variant rs6265.

Previous studies have shown that men, older age, higher BMI, diabetes, singles, and a lower education level were associated with higher stress levels related to high nicotine dependence^4,5^. Consequently, when providing anti-smoking treatment to patients with these clinical, psychological, and socioeconomic statuses, it is essential to evaluate their nicotine dependence^5^. Additionally, longitudinal cohort studies should be conducted to determine the causal relationship between nicotine dependence and these clinical, psychological, and socioeconomic factors^5^. Moreover, a previous study has highlighted the correlation between successful smoking cessation and marital status and health behaviours among middle-aged smokers, particularly men^6^. Qiu and colleagues explored factors affecting smoking cessation by age group among Chinese adult male smokers^7^. Results showed that successful smoking cessation programs were mainly associated with older age, lower nicotine dependence, and self-reported poor health^7^. Kim and his colleagues observed that the different factors associated with the success or failure of smoking cessation varied by age^8^. However, different strategies to help in these effort may need to be tailored to individuals’ geographic location, gender, and ethnicity/race^9^.

More recently, literature has offered contradictory findings on the correlations between rs6265 and smoking cessation behaviors. One study concluded that a person who carries the Met allele of BDNF missense polymorphism would initiate and maintain smoking behavior^10^. In contrast, a meta-analysis published by the tobacco and genetics consortium^11^ suggests the strongest association was between nonsynonymous SNP in BDNF and chromosome 11 (rs6265). Two studies conducted among Asian populations^12^ indicated reduced nicotine dependence among current smokers who carried the BDNF Met allele relative to those homozygous for the Val allele. Despite BDNF Val66Met having no direct effect on smoking cessation^13^, Ohmoto and Takahashi demonstrated that this polymorphism influenced the smoking initiation age, suggesting BDNF polymorphism could enhance the reinforcing effects of nicotine in some way^13^ These results were similar to the findings by Xiang and colleagues who investigated the relationship between BDNF Val66Met variant and smoking in a Chinese population^14^. The present data indicates that carriers of the BDNF Met allele might be more vulnerable to lifetime cigarette smoking and also cigarette smoking within 12 months^15^. Su and colleagues used Taiwan Biobank dataset investigated smoking behaviour in specific populations, individuals living with schizophrenia^16^. However, the evidence regarding the influence of rs6265 on smoking behaviour in general population remains inconsistent.

Nevertheless, replication and extension of the current findings that BDNF is a potential locus for smoking initiation could further improve our understanding of the issue and enhance effective preventative therapies for those with specific genetic variants^14^. Previous meta-analyses showed that the Val66Met variant has a moderate effect on smoking persistence in a large-scale sample. When compared among individuals with the Met/Met genotype, individuals with the Val/* genotype had a 23% greater possibility of maintaining smoking^17^. Previous research conducted in the Finnish population found a robust and independent association between BDNF Val66Met and smoking initiation (SI), with results suggesting that this association is not influenced by depression^18^.

While some evidence suggests significant associations between socio-demographic, lifestyle factors, the rs6265 polymorphism and smoking cessation behaviours, further examination in different populations is required to confirm these findings. We examined the role of rs6265 TT, CC and TC genotypes in association with socio-demographic and other variables, including mental health status, drinking, exercise, and smoking behaviour among Taiwanese adults.

## Results

The descriptive data of 20,584 participants are presented in Table 1. Significant differences were found between current and former smokers for sex, age, drinking behaviour, education level, exercise, body mass index (BMI), marital status, and education level (*P*-value < 0.0001). These findings suggest that genetic, social, and environmental factors play a role in smoking persistence. There were no significant differences for the rs6265 genotypes, depression, and bipolar disorder. The polymorphism rs6265 did not show a significant difference between current and former smokers (*P*-value = 0.8753). The sex variable was significantly different (*P*-value < 0.0001) and all the other confounders were significantly different between current and former smokers in the analysis.

**Table 1:** Demographic characteristics of current smokers and former smokers.

Variables	Current smoker	Former smoker	*P*-value
	(N = 9641)	(N = 10,943)	
rs6265 n, %			0.8753
TT	2408 (24.98)	2767 (25.29)	
TC	4840 (50.20)	5476 (50.04)	
CC	2393 (24.82)	2700 (24.67)	
Sex n, %			< .0001
Female	1899 (19.70)	1662 (15.19)	
Male	7742 (80.30)	9281 (84.81)	
Age (years)	47.159 ± 0.104	52.490 ± 0.098	< .0001
Drinking n, %			< .0001
No	6627 (68.74)	8165 (74.61)	
Yes	3014 (31.36)	2778 (25.39)	
Exercise n, %			< .0001
No	6896 (71.53)	5569 (50.89)	
Yes	2745 (28.47)	5374 (49.11)	
BMI (kg/m^2^) n, %			< .0001
Underweight	252 (2.61)	122 (1.11)	
Normal	3576 (37.09)	3846 (35.15)	
Overweight	2943 (30.53)	3880 (35.46)	
Obese	2870 (29.77)	3095 (28.28)	
Marital status (n), %			< .0001
Unmarried	1532 (15.89)	874 (7.99)	
Married	6582 (68.27)	8991 (82.16)	
Divorced or separated	1364 (14.15)	883 (8.07)	
Widowed	163 (1.69)	195 (1.78)	
Education level (n), %			< .0001
Primary school and under	313 (3.25)	433 (3.96)	
Junior and senior school	4545 (47.14)	4391 (40.13)	
University and above	4783 (49.61)	6119 (55.92)	
Depression n, %			0.0027
No	9211 (95.54)	10,545 (96.36)	
Yes	430 (4.46)	398 (3.64)	
Bipolar disorder n, %			0.0041
No	9523 (98.78)	10,853 (99.18)	
Yes	118 (1.22)	90 (0.82)	

Table 2 shows the association between current and former smokers and sociodemographic factors based on logistic regression. Men showed a significantly lower probability of quitting smoking than women (OR = 0.750, 95% CI 0.689–0.817). Participants with drinking habits also appeared to have a decreased probability of quitting smoking (OR = 0.707, 95% CI 0.662–0.754). Former smokers had higher probabilities of exercise habits (OR = 1.829, 95% CI 1.718–1.947) while increased age showed a similar trend (OR = 1.045, 95% CI 1.041–1.048). Moreover, results showed that married couples were affected exhibited smoking behaviour when compared to the unmarried group. The Divorced or separated group had more current smokers (OR = 0.763, 95% CI 0.671–0.867), and more former smokers were married (OR = 1.535, 95% CI 1.393–1.691).

**Table 2:** Multivariable logistic regression analysis showing the association between social-demographic factors, lifestyle, mental health, and smoking status.

Variables	OR	95% CI	*P*-value
Sex			
Women	1		
Men	0.750	0.689–0.817	< .0001
Age (years)	1.045	1.041–1.048	< .0001
Drinking			
No	1		
Yes	0.707	0.662–0.754	< .0001
Exercise			
No	1		
Yes	1.829	1.718–1.947	< .0001
BMI			
Normal	1		
Underweight	0.597	0.473–0.754	< .0001
Overweight	1.131	1.054–1.215	0.0006
Obese	1.095	1.018–1.178	0.0147
Marital status			
Unmarried	1		
Married	1.535	1.393–1.691	< .0001
Divorced or separated	0.763	0.671–0.867	< .0001
Widowed	0.943	0.742–1.198	0.6293
Education level			
Primary school and under	1		
Junior and senior school	1.162	0.990–1.366	0.0668
University and above	1.541	1.311–1.813	< .0001
Depression			
No	1		
Yes	0.941	0.809–1.095	0.4321
Bipolar disorder			
No	1		
Yes	0.815	0.607–1.095	0.1741

The comparison of the rs6265 genotypes is shown in Table 3. BDNF rs6265 TC and CC genotypes did not reach statistical significance when compared to the TT genotype, the p-values were 0.6629 and 0.3913, respectively. The trends of other confounders were the same as shown in Table 2. Higher BMI levels, such as overweight and obesity, were associated with a higher probability of smoking cessation. The odds ratios were 1.132 (95% CI 1.054–1.215) and 1.096 (95% CI 1.018–1.178). On the contrary, smoking cessation seemed more challenging to underweight subjects (OR = 0.597, 95% CI 0.473–0.754). Participants with university education and above had a significantly higher probability of quitting smoking compared to those with primary education and under (OR = 1.542, 95% CI = 1.311–1.813).

**Table 3:** Multivariable logistic regression analysis showing the association between social demographic factors, lifestyle, mental health, and smoking status when adjusted for rs6265.

Variables	OR	95% CI	*P*-value
rs6265			
TT	1		
TC	0.984	0.917–1.057	0.6629
CC	0.965	0.889–1.047	0.3913
Sex			
Women	1		
Men	0.750	0.688–0.816	< .0001
Age (years)	1.045	1.041–1.048	< .0001
Drinking			
No	1		
Yes	0.707	0.662–0.754	< .0001
Exercise			
No	1		
Yes	1.830	1.719–1.947	< .0001
BMI			
Normal	1		
Underweight	0.597	0.473–0.754	< .0001
Overweight	1.132	1.054–1.215	0.0006
Obese	1.096	1.018–1.179	0.0145
Marital status			
Unmarried	1		
Married	1.534	1.392–1.691	< .0001
Divorced or separated	0.762	0.671–0.866	< .0001
Widowed	0.942	0.741–1.197	0.6229
Education level			
Primary school and under	1		
Junior and senior school	1.162	0.990–1.366	0.0668
University and above	1.542	1.311–1.813	< .0001
Depression			
No	1		
Yes	0.943	0.810–1.097	0.4434
Bipolar disorder			
No	1		
Yes	0.814	0.607–1.094	0.1723

## Discussion

In our study, we found an association between sex, exercise, drinking, weight, marital status, and smoking cessation behaviour in the Taiwanese adult population. Under-weight, male, and divorced individuals, alongside regular drinkers were more prone to continued smoking. In contrast, older persons with exercise habits and overweight persons with higher education levels were more likely to quit smoking. However, the two observed variables, mental health-depression and bipolar disorder were not significant. Contrary to expectations, this study did not find significant results for smoking cessation behaviour among carriers and non-carriers of the BDNF rs62625.

Sex and age have been the most researched unmodifiable factors associated with smoking cessation behaviour. A previous study underscores the association of successful smoking cessation with changes in job status and health behaviours among middle-aged smokers, especially men^6^. Our study results mirror those of previous studies that examined both sexes^6,19^. In our study, divorced males found it more challenging to quit smoking. In accordance with the present results, previous studies have shown that despite a significant decrease in mortality rates among non-smokers over the past 50 years due to disease prevention and improved medical treatment, the smoker vs. non-smoker death rate ratio has increased substantially due to earlier and more intensive use of cigarettes, which has negated the decreased mortality rates^20^. Cessation at age 50 halved the hazard among cigarette smokers, and cessation at age 30 avoided almost all of it^20^. This study confirms that the older aged population is more likely to quit smoking, which is consistent with a follow-up study (cohort study) of over 5 years^7^. In previous findings, low education level was found to be related to higher nicotine dependence^5^. In another study, however, smoking cessation behaviours were not associated with education level^7^. In our study, participants with university degrees were higher among the non-smoker group.

Lifestyle behaviours including drinking and exercise were shown to be predictive factors for individual who quit smoking behaviour. These results reflect those of Oshio who also found that it was easier for persons without drinking habits to quit smoking^6^. A smoke-free environment, willpower and abstinence from alcohol are crucial to the success of smoking cessation^9^. Previous study found that the efficacy of exercise as an adjunct to smoking cessation treatment was found to be particularly effective among high-anxiety sensitive smokers who were Val/Val carriers^21^. This finding is consistent with results reported on the role of BDNF as a mediator and successful factor in smoking cessation program among smokers who were Val/Val carriers^21^. These data encourage further evaluation of the association between BDNF polymorphism, exercise, anxiety sensitivity and smoking cessation. This study also supports evidence from the clinical observations which show that higher BMI related to high nicotine dependence was linked to successful quitting among participants aged 40 years and above^5,8^. Studies conducted in Asian populations found that BDNF rs6265 was related to BMI. It was found that Met carriers appeared to have lower BMI, similar to the findings in Caucasians. The smoking behaviour among Met carriers was found to be correlated with their BMI^12^. Consistent with previous studies^9^, when we compared groups with different BMI, we found that underweight populations were more likely to continue smoking.

It has been suggested that current smokers have higher levels of BDNF than non-smoking individuals, not due to nicotine dependence and/or BDNF Val66Met poly-morphism^22^. Moreover, higher serum BDNF was found to be positively associated with chronic cigarette smoking but there is limited evidence on the association with smoking cessation behaviours. Xia found a weak association between BNDF level or the BDNF Val66Met polymorphism and smoking, as well as a significantly negative correlation between serum BDNF level and attention, and a positive correlation between BDNF level and CO concentration in the smoker group^23^. Furthermore, the associations observed in smokers were only seen in Val allele carriers, suggesting that they may be dependent on the BDNF Val66Met polymorphism. With a larger sample size and cross-sectional design, a previous study reiterated the interrelationships between smoking and BDNF^24^. However, this does not appear to be the case. The present analysis indicate that carriers of the BDNF Met allele might not be more vulnerable to quitting smoking than non-carriers, which is consistent with findings from an Iranian population^15^.

Additionally, a previous meta-analyses showed that the Val66Met variant moderately affects smoking persistence in a large-scale sample, and the possibility of continued smoking was 23% greater for individuals with the Val/* genotype compared to those with the Met/Met genotype, indicating that the dopamine-relevant functions of the Val66Met variant may be involved in the biological process associated with smoking persistence^17^. An earlier study reported the association between BDNF Val66Met and SI in a Finnish population^18^. The effect size was similar to that reported in the 2010 large meta-analyses. In a Finnish population data suggest that the association of BDNF with smoking initiation is robust and independent of depression^18^. Previous studies regarding the relationship between BDNF Met allele carriers and smoking behaviour have shown varied results, ranging from positive and negative to non-significant associations. Recent research suggests that there may be genetic presentation to certain social and environmental factors^25^. In this study, we focused on the Taiwanese population. We assumed that BDNF Met allele carriers are more likely to continue smoking, as past studies have linked this gene to nicotine addiction. Finally, we collected data from 20,584 participants and analysed their genetic, sociodemographic and lifestyle data in association with their smoking cessation behaviour. We included individuals who were genotyped and completed self-reported socio-demographic and lifestyle behavior queationnaires, with a large sample size for a biomarker study. However, a certain gender imbalance between current and former smokers was evident. Exclusion of individuals who quit smoking for less than 6 months and those who were nicotine dependent from this study is considered a limitation.

## Conclusion

This study examined the rs6265 TT, CC and TC genotypes and their association with socio-demographic and other variables. We found that genetic variants associated with smoking persistence are shared across different ethnic groups. Future studies should use GWAS with biomarkers or longitudinal assessments to refine phenotypic assessments, and should include multiple ethnic groups to further investigate the genetics of smoking.

## Methods

### Data source and participants

The participants’ data were obtained from the Taiwan Biobank (TWB), which dates back to 2008. Taiwanese participants, aged between 30 and 70 years, without a history of cancer diagnosis were enrolled into the TWB after signing an informed consent form. Participants completed a self-reported questionnaire, physical measurements, and urine/blood collection during the registration period. A total of 132,720 participants were enrolled from 2008 to 2019. After excluding samples with missing genotypes (n = 32,343), never smokers (n = 79,679) and missing values in the regression model (n = 114), a total of 20,584 subjects were retained for analyses. The study was approved by the Institution Review Board of Chung Shan Medical University Hospital (with IRB approval ID: CSI-20009). All authors confirmed that all methods were performed in accordance with the relevant guidelines and regulations.

### Assessment of variables

The data on Brain-Derived Neurotrophic Factor (BDNF) single nucleotide polymorphism rs6265 in the current study came from genotyping. Based on the TWBv1.0 and TWBv2.0 chips, we retrieved data from 100,377 participants. The genotyping information passed quality control., The rs6265 SNP had a higher call rate and did not deviate from the Hardy–Weinberg equilibrium. Its minor allele frequency was >0.05. Information on smoking status was collected from a questionnaire. If participants smoked for more than 6 months prior to enrolment, they were defined as currently smoking. With regard to smoking behavior, current smokers were defined as those who were smoking prior to assessment and had been smoking for at least 6 months. Smoking cessation behavior was defined as having smoked for at least 6 months but later quit at least 6 months before assessment at the biobank centers. Exclusion criteria included non- or occasional smokers (absence of habitual smoking, for a duration of less than 6 months). Body mass index (BMI) was divided into four groups as defined by the Health Promotion Administration: normal (18.5 ≤ BMI < 24), underweight (BMI < 18.5), 24 ≤ BMI < 27 (overweight) and BMI ≥ 27 (obese). Information on drinking, exercise, depression and bipolar disorder were all collected from the questionnaires and were categorized as yes/no. Drinking referred to a weekly alcohol intake of 150c.c. for six consecutive months. Regular exercise habit referred to exercising at least three times a week, for at least 30 min each time.

### Selection of the rs6265 genetic variant

Chauhan published an updated review on single nucleotide polymorphisms of the Brain-derived Neurotrophic Factor (BDNF) associated with obesity, hypertension, short-term plasticity and learning^23^. Previous studies suggested that longitudinally changes in BDNF in incident smokers and persons who quit smoking must be addressed to better understand the nature of the relationship between smoking and serum BDNF concentrations^21^. This paper attempts to examine separate genetic locus that contribute modestly to phenotypic variability in each aspect of smoking behaviour, which, in turn, may have implications for how smoking cessation therapies and tobacco control efforts are designed and targeted^10,11,14^. Notably, the five significant loci identified in previous meta-analyses were each associated with only one specific smoking phenotype. Since Lin suggested that independent replication studies with larger sample sizes could provide further insights into the role of the BDNF gene, we conducted a thorough examination of the relationship between BDNF and psychological distress in Taiwanese populations^26^. Our results suggested that BDNF genetic variants may be linked to psychological distress both independently and through SNP-SNP and gene-physical activity interactions.

### Statistical analysis

We employed the statistical analysis system (SAS) version 9.4 (SAS Institute, Cary, NC, USA) to determine the association between rs6265 and smoking behaviour. Plink 1.9 software was used to analyze genotype/phenotype data. The statistically significant value in the study was set at 0.05. The Chi-square test was used to examine differences between categorical variables (rs6265, sex, drinking, and exercise were presented as number and percentage). Student’s t-test was used to compare differences in means between continuous variables. Results were presented as mean ± standard deviation. In order to examine the relationship between categorical variables, we used a multivariate logistic regression model to estimate the Odds ratios (ORs) and 95% confidence intervals (CIs) associated with smoking behavior (Supplementary Table 1).

### Supplementary Information

Supplementary Table 1.

## Data Availability

The data used in this study are available from the Taiwan Biobank dataset. The usage of these data is restricted under the regulation set by the authority of the Taiwan Biobank dataset, so these data are not publicly available. Data are however available from the corresponding authors upon reasonable request and with permission of Taiwan Biobank dataset.
