# Supplementary material for: The association between smoking cessation and lifestyle/genetic variant rs6265 among the adult population in Taiwan
Source: Sci Rep. 2023 Dec 18;13:22622. doi: 10.1038/s41598-023-48806-x (PMC10730844; doi:10.1038/s41598-023-48806-x)
Supplement: Supplementary file 1 — Supplementary Table 1. [file 41598_2023_48806_MOESM1_ESM.docx]

Table 1. Demographic characteristics stratified by rs6265

| Variables | rs6265 TT | rs6265 TC | rs6265 CC | P-value |
| --- | --- | --- | --- | --- |
|  | (n = 5175) | (n = 10316) | (n = 5093) |  |
| Cigarette smoking status n, % |  |  |  | 0.8753 |
| Current smoker | 2408 (46.53) | 4840 (46.92) | 2393 (46.99) |  |
| Former smoker | 2767 (53.47) | 5476 (53.08) | 2700 (53.01) |  |
| Sex n, % |  |  |  | 0.0111 |
| Female | 833 (16.10) | 1795 (17.40) | 933 (18.32) |  |
| Male | 4342 (83.90) | 8521 (82.60) | 4160 (81.68) |  |
| Age (years) | 49.967 ± 0.145 | 50.015 ± 0.104 | 49.976 ± 0.150 | 0.9573 |
| Drinking n, % |  |  |  | 0.0745 |
| No | 3721 (71.90) | 7352 (71.27) | 3719 (73.02) |  |
| Yes | 1454 (28.10) | 2964 (28.73) | 1374 (26.98) |  |
| Exercise n, % |  |  |  | 0.3628 |
| No | 3176 (61.37) | 6228 (60.37) | 3061 (60.10) |  |
| Yes | 1999 (38.63) | 4088 (39.63) | 2032 (39.90) |  |
| BMI (kg/m^2^) n, % |  |  |  | 0.2506 |
| Normal | 1829 (35.34) | 3812 (36.95) | 1781 (34.97) |  |
| Underweight | 96 (1.86) | 181 (1.75) | 97 (1.90) |  |
| Overweight | 1741 (33.64) | 3358 (32.55) | 1724 (33.85) |  |
| Obese | 1509 (29.16) | 2965 (28.74) | 1491 (29.28) |  |
| Marital status (n), % |  |  |  | 0.3546 |
| Unmarried | 573 (11.07) | 1217 (11.80) | 616 (12.10) |  |
| Married | 3966 (76.64) | 7760 (75.22) | 3847 (75.54) |  |
| Divorced or separated | 549 (10.61) | 1146 (11.11) | 552 (10.84) |  |
| Widowed | 87 (1.68) | 193 (1.87) | 78 (1.53) |  |
| Education level (n), % |  |  |  | 0.5508 |
| Primary school and under | 187 (3.61) | 376 (43.97) | 183 (3.59) |  |
| Junior and senior school | 2229 (43.07) | 4536 (43.97) | 2171 (42.63) |  |
| University and above | 2759 (53.31) | 5404 (52.38) | 2739 (53.78) |  |
| Depression n, % |  |  |  | 0.0014 |
| No | 5007 (96.75) | 9892 (95.89) | 4857 (95.37) |  |
| Yes | 168 (3.25) | 424 (4.11) | 236 (4.63) |  |
| Bipolar disorder n, % |  |  |  | 0.6028 |
| No | 5117 (98.88) | 10218 (99.05) | 5041 (98.98) |  |
| Yes | 58 (1.12) | 98 (0.95) | 52 (1.02) |  |
